# Gastric microbiota and predicted gene functions are altered after subtotal gastrectomy in patients with gastric cancer

**DOI:** 10.1038/srep20701

**Published:** 2016-02-10

**Authors:** Ching-Hung Tseng, Jaw-Town Lin, Hsiu J. Ho, Zi-Lun Lai, Chang-Bi Wang, Sen-Lin Tang, Chun-Ying Wu

**Affiliations:** 1 Bioinformatics Program, Taiwan International Graduate Program, Academia Sinica, Taipei 11529, Taiwan; 2 Biodiversity Research Center, Academia Sinica, Taipei 11529, Taiwan; 3 Institute of Biomedical Informatics, National Yang-Ming University, Taipei 11221, Taiwan; 4 School of Medicine, Fu Jen Catholic University, New Taipei City 24205, Taiwan; 5 Institute of Population Health Sciences, National Health Research Institutes, Miaoli 35053, Taiwan; 6 Division of Gastroenterology, Taichung Veterans General Hospital, Taichung 40705, Taiwan; 7 Department of Public Health, China Medical University, Taichung 40402, Taiwan; 8 Graduate Institute of Clinical Medical Sciences, China Medical University, Taichung 40402, Taiwan; 9 Faculty of Medicine and Graduate Institute of Clinical Medicine, National Yang-Ming University, Taipei 11221, Taiwan; 10 National Institute of Cancer Research, National Health Research Institutes, Miaoli 35053, Taiwan; 11 Department of Life Sciences and Rong Hsing Research Center for Translational Medicine, National Chung-Hsing University, Taichung 40227, Taiwan

## Abstract

Subtotal gastrectomy (*i.e.*, partial removal of the stomach), a surgical treatment for early-stage distal gastric cancer, is usually accompanied by highly selective vagotomy and Billroth II reconstruction, leading to dramatic changes in the gastric environment. Based on accumulating evidence of a strong link between human gut microbiota and host health, a 2-year follow-up study was conducted to characterize the effects of subtotal gastrectomy. Gastric microbiota and predicted gene functions inferred from 16S rRNA gene sequencing were analyzed before and after surgery. The results demonstrated that gastric microbiota is significantly more diverse after surgery. *Ralstonia* and *Helicobacter* were the top two genera of discriminant abundance in the cancerous stomach before surgery, while *Streptococcus* and *Prevotella* were the two most abundant genera after tumor excision. Furthermore, *N*-nitrosation genes were prevalent before surgery, whereas bile salt hydrolase, NO and N_2_O reductase were prevalent afterward. To our knowledge, this is the first report to document changes in gastric microbiota before and after surgical treatment of stomach cancer.

Advances in sequencing technologies and analytical methods have enabled characterization of the human gut microbiota. As part of the human gut, the stomach is inhabited by a wide variety of bacteria, despite the long-held notion of it being a hostile environment for microbial colonization^1^,^2^. In healthy individuals, several genera other than *Helicobacter*, including *Streptococcus, Prevotella, Veillonella, Rothia*, and *Neisseria*, are abundant in the stomach, based on cloning^3^,^4^ and pyrosequencing^5^,^6^. Regarding effects of diseases, the gastric microbiota shifts towards decreasing diversity with progression of gastritis, intestinal metaplasia, and gastric cancer^7^. However, the gastric microbiota of elderly stomach cancer patients does not significantly differ from that of dyspeptic controls^8^. Although chronic infection with *Helicobacter pylori* causes serious gastric complications, there are no significant associations between microbial phylotypes and *H. pylori* status of the stomach^3^. In that regard, *H. pylori* status only explains 28% of the variance in gastric microbiota, whereas 44% is explained by host factors^9^. Understanding of human gastric microbiota is in its infancy and is complicated by changes over time in persons with complicated gastric syndromes (which require long-term follow-up and invasive sampling).

Subtotal gastrectomy (*i.e.*, partial removal of the stomach) is a surgical treatment for distal gastric cancer, a multifactorial disease causing numerous cancer-related deaths around the world^10^. Patients receiving subtotal gastrectomy for gastric cancer are often subjected to other surgical procedures, which alter the gastric environment. For example, highly selective vagotomy affects gastric secretion of gastric acid^11^; cholecystectomy elevates gastric pH value^12^; Billroth II reconstruction reduces pancreatic polypeptide secretion^13^. Following subtotal gastrectomy, there are several common side effects, including marginal ulcers, bile reflux, and stump cancer. Bile reflux after subtotal gastrectomy has been associated with the presence of *Streptococcus* and *Veillonella* in gastric aspirates^14^ and *Escherichia, Klebsiella*, and *Clostridium* in the intestine^15^. Although gastric microbiota is altered after subtotal gastrectomy^14^, changes in diversity have not been well characterized. Despite characterization of gastric microbiota by culture-independent approaches, changes following subtotal gastrectomy in patients with gastric cancer are not completely understood. Therefore, there are many knowledge gaps, leading to a number of questions. For example, are different anatomic sites inhabited by different microbes? What is the compositional variation in gastric microbiota after subtotal gastrectomy? What is the biodiversity pattern before and after subtotal gastrectomy? Do metabolic functions embedded in gastric microbiota correspond to changes caused by subtotal gastrectomy? In this study, we aimed to address these questions by deep sequencing of microbial 16S ribosomal RNA (rRNA) genes in gastric tissues.

Gastric microbiota in gastric cancer patients (at various anatomic sites and before and after subtotal gastrectomy) was characterized by 16S rRNA gene sequencing. Within the 2-year timeframe of this follow-up study, 24 gastric biopsies were collected from 6 patients subjected to subtotal gastrectomy. Variations in gastric microbiota and predicted gene functions before and after tumor excision (subtotal gastrectomy) were determined.

## Results

### Statistical summaries of sequencing results

To characterize stomach bacterial microbiota and potential variations associated with subtotal gastrectomy, we collected tumor (abbreviated as T in figures) and non-tumor (N) tissues before surgery, as well as gastric stump (S) and high body (B) tissues after surgery, from 6 gastric cancer patients. A total of 4.6 million pair-end reads were generated, of which 3.2 million reads passed quality filtering and were non-chimeric. To determine bacterial community diversity and composition, reads were aligned to the Greengenes database and non-bacterial sequences removed. On average, 85% of reads in a sample were retained. In total, 2.7 million reads (on average, 113 ± 42 thousand reads per sample) were used for subsequent analyses.

### Bacterial diversity

Based on 16S rRNA gene sequencing data, bacterial richness (number of operational taxonomic units (OTUs), richness value, and Chao 1 index) increased after surgery (*P* < 0.01, one-tailed Student’s *t*-test; Table 1). Similarly, there was greater diversity of bacterial communities after surgery (Shannon index) than before surgery (*P* < 0.01, one-tailed Student’s *t*-test; Table 1). However, within patients, there were no differences (*P* > 0.05, two-tailed paired *t*-test of Shannon indices) between tumor and non-tumor tissues (before surgery) or between gastric stump and high body tissues (after surgery). Before-surgery communities were characterized by rarefaction curves approaching an asymptote, whereas after-surgery communities were characterized by curves with steeper slopes (Figure S1), indicating the potential for greater diversity to be discovered with more sequencing efforts. Good’s coverage estimates sampling completeness by calculating the probability that a randomly selected read from a sample has been sequenced. At 97% sequence similarity level, Good’s coverage values for all sampled bacterial communities ranged from 0.964 to 0.996 (except T594S, with 0.893 Good’s coverage) when estimated using all reads (Table S1).

### Bacterial community structure

**Figure 1:**
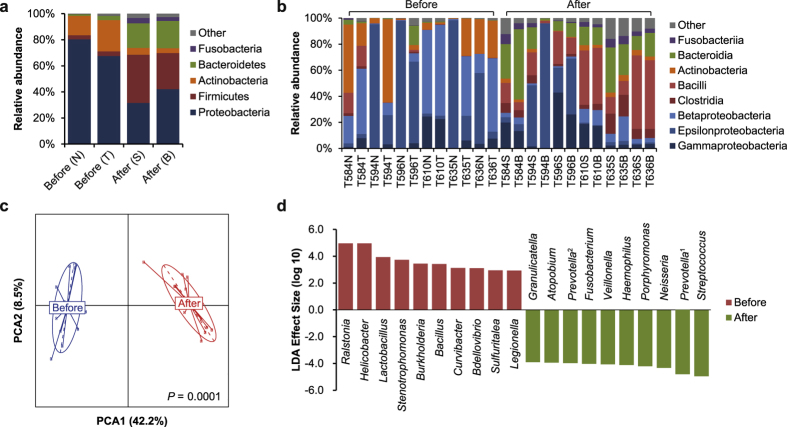
Bacterial community composition of human stomach, before and after surgery. **(a)** Average relative abundance of phyla across all samples, divided by tissue type and surgery status. **(b)** Relative abundance of classes across all samples. **(c)** Principal component analysis of bacterial genera abundance, with before and after surgery serving as instrumental variable. **(d)** Top-10 known genera with the highest LDA effect sizes reported by LEfSe in the stomach bacterial community, before and after surgery. ^1^This *Prevotella* genus is affiliated with Prevotellaceae. ^2^This *Prevotella* genus is affiliated with Paraprevotellaceae, a recommended family (based on the Greengenes database).

Bacterial abundance (*i.e.*, read count) was normalized by corresponding copy number of 16S rRNA genes (prior to community compositional analysis) to reduce potential bias of abundance estimation due to copy number variation^16^. Before surgery, stomach microbiota in tumor and non-tumor tissues was dominated by the phyla Proteobacteria (80% in N and 67% in T) and Actinobacteria (15% in N and 24% in T), followed by Firmicutes and Bacteroidetes (<4% in both N and T; Fig. 1A). However, after surgery, Firmicutes and Bacteroidetes dramatically increased in both gastric stump and high body tissues (32 and 20% respectively, on average), while Proteobacteria and Actinobacteria decreased (Fig. 1A).

At the class level, there was obvious personal variation in stomach microbiota. For example, in 3 before-surgery samples Epsilonproteobacteria was dominant (>95%), whereas 5 samples harbored Actinobacteria (at least 25%; Fig. 1B). After surgery, Bacilli (of Firmicutes) and Bacteroidia (of Bacteroidetes) significantly increased in the stomach of most patients, representing (on average) 25 and 19% of the microbiota, respectively.

Principal component analysis (PCA) with surgery status as instrumental variable revealed significant differences in bacterial genera abundance before and after surgery (*P* = 0.0001, Monte-Carlo simulation; Fig. 1C). Furthermore, from the results of linear discriminant analysis (LDA) effect size (LEfSe) analysis^17^, there were a total of 63 known genera with differential abundance before and after surgery. Among 19 genera known to be more abundant before surgery, *Ralstonia* and *Helicobacter* were the two with the largest LDA effect size (Fig. 1D), whereas *Streptococcus* and *Prevotella* represented the top two genera (of 43) after surgery.

**Figure 2:**
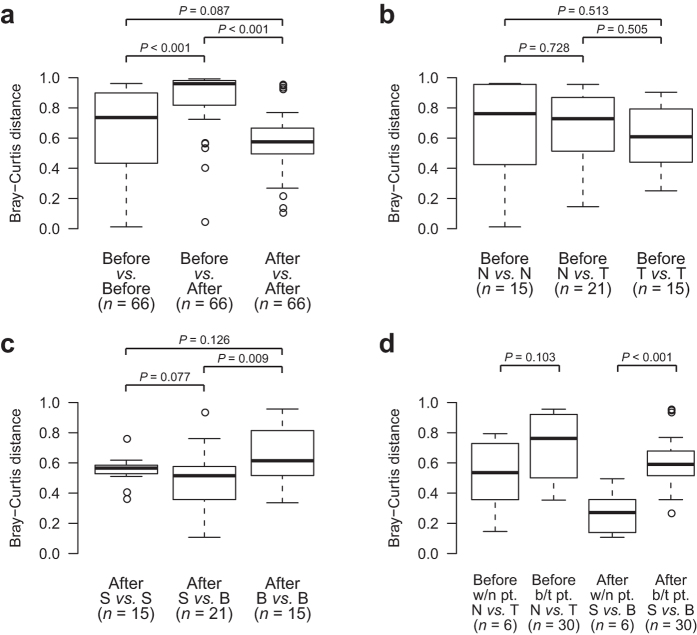
Bacterial community structure dissimilarity summary. Dissimilarity was measured using Bray-Curtis distance of beta diversity (genus level) and group difference was determined (two-tailed Wilcoxon rank-sum test, with significance and sample size N noted on the figure). **(a)** Comparison of dissimilarity values among communities before and after surgery. **(b)** Comparison of dissimilarity values among communities in tumor and non-tumor tissues before surgery. **(c)** Comparison of dissimilarity values among communities in gastric stump and high body tissues after surgery. **(d)** Comparison of dissimilarity values among communities within and between patients, before and after surgery. Abbreviations: w/n, within; b/t, between; pt., patient.

Group differences (*i.e.*, before and after surgery) in bacterial community structure were also observed using the Bray-Curtis measure of beta diversity^18^. The within-group distance was significantly lower than the between-group distance when samples were divided into before- and after-surgery groups (*P* < 0.001; Fig. 2A). However, comparisons between tumor and non-tumor tissues before surgery (Fig. 2B) and between gastric stump and high body tissues after surgery (Fig. 2C) showed insignificant or only marginally significant differences in distances. Interestingly, community dissimilarity within patients was lower than that between patients (regardless of surgery status), although only after-surgery communities demonstrated a significant difference (Fig. 2D).

### Bacterial gene functions

Based on the community structure derived from 16S rRNA gene sequencing data, predicted gene functions of each sample were inferred from referenced bacterial genomes^19^. Enrichment analysis was performed (two-group comparison^20^) to identify gene functions of differential abundances that were significantly enriched (Benjamini-Hochberg adjusted *P* < 0.05) in bacterial microbiota before and after surgery. In agreement with the dramatically changed community composition, there were 1879 and 2394 Clusters of Orthologous Groups (COGs) enriched in samples before (Table S2) and after surgery (Table S3), respectively. Given that there were so many genes of differential abundance, and based on the understanding of metabolic diversity of gut microbiota^21^,^22^, functional genes related to gastric disease and carcinogenesis were prioritized for further investigation.

**Figure 3:**
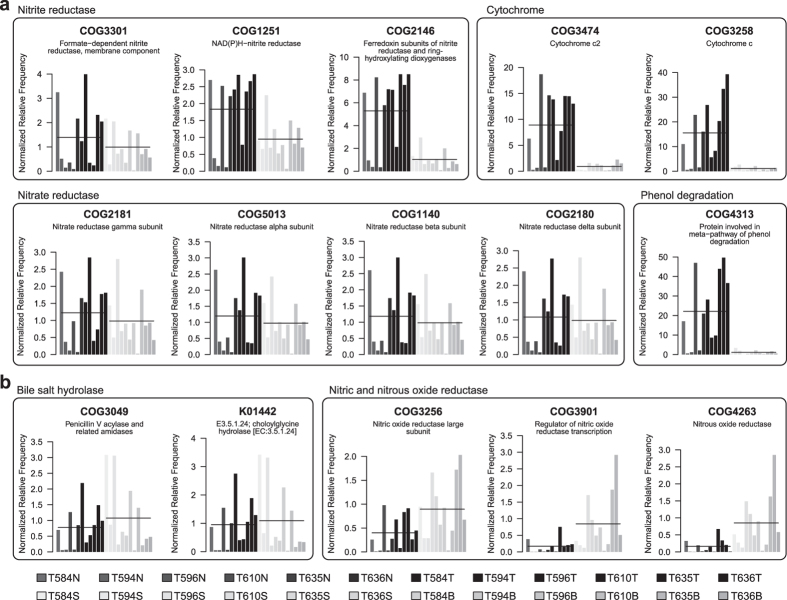
Abundance of predicted gene families differentially enriched in the gastric microbiota before and after surgery. Significance was defined as adjusted *P* < 0.05. **(a)** Predicted gene functions enriched in the before-surgery microbiota. **(b)** Predicted gene functions enriched in the after-surgery microbiota. Horizontal lines indicate group means (based on normalized relative frequencies).

Metabolic enzymes involved in denitrification (*e.g.*, nitrate and nitrite reductases) were more abundant in the gastric microbiota before surgery (Fig. 3A). Furthermore, genes related to nitrosation (conversion of organic compounds to nitroso derivatives) were also more abundant before surgery, including the gene families of phenol degradation (Fig. 3A; COG4313) and cytochrome (COG3474 and COG3258). From pathway-centric comparisons, biosynthesis of various vitamins, including biotin (vitamin H), riboflavin (vitamin B12), pyridoxal phosphate (vitamin B6), cobalamin (vitamin B12), thiamine (vitamin B1), and menaquinone (vitamin K), was more prevalent in the gastric microbiota before surgery than after surgery (Table S4). Conversely, after-surgery gastric microbiota encoded more bile salt hydrolase (Fig. 3B; COG3049 and K01442) and genes related to reduction of nitric oxide (NO) and nitrous oxide (N_2_O). There were 36 COG Pathways enriched in the gastric microbiota after surgery (Table S5).

## Discussion

This is the first follow-up study in which deep sequencing is applied to investigate the stomach microbiota in gastric cancer patients before and after subtotal gastrectomy. This study is also the first to predict gene functions associated with variations in gastric microbiota. There was an obvious shift in community composition of the gastric microbiota after surgery. Decreases in Proteobacteria and Actinobacteria and increases in Firmicutes and Bacteroidetes defined the after-surgery microbiota shift (at phylum level). *Ralstonia* and *Helicobacter* were the top two genera of discriminant abundance in the stomach of patients with gastric cancer, while *Streptococcus* and *Prevotella* were the top two genera after surgery. In contrast, there was relatively low divergence of gastric microbiota among various sites in the stomach. Corresponding to the community shift, the gastric microbiota also exhibited differential predicted gene functions. For example, denitrification and nitrosation genes were prevalent in patient stomachs before surgery, whereas bile salt hydrolase and NO and N_2_O reductases were prevalent after surgery.

After gastrectomy, the stomach was dominated by only four phyla (Proteobacteria, Firmicutes, Bacteroidetes, and Actinobacteria), which are in abundance in the gastric microbiota of healthy individuals^3^,^5^. The relative abundance of these phyla covaries with *H. pylori* status^9^. Composition of the gastric microbiota gradually changes along with progression of gastric diseases (from gastritis to intestinal metaplasia and ultimately gastric cancer^7^,^23^). This study extended observations corresponding to surgical removal of gastric cancer tissue, resulting in increases in Firmicutes and Bacteroidetes and decreases in Actinobacteria and Proteobacteria.

In the present study, gastric cancer tissue and neighboring normal tissue demonstrated similar microbiota. After subtotal gastrectomy, the within-patient microbiota revealed even greater similarity between gastric stump and high body tissues, similar to the findings of a previous report in which there was little difference in gastric microbiota between antrum and body biopsy specimens^4^. Based on cultures of biopsy specimens, 62% of gastric microbiota exist in both the antrum and body of the stomach^24^, which is as expected as these two sites are distinct niches for microbial colonization, given their differential ability to secrete gastric acid^25^. Based on the present results and those of previous studies, we inferred that the gastric environment as a whole is more critical than the individual anatomical sites in determining microbial composition.

Gastric acidity is a barrier to microbial overgrowth^26^,^27^. However, the secretion of gastric acid is reduced after vagotomy^11^ (*i.e.*, cutting branches of the vagus nerve), resulting in a mildly acidic stomach that allows for more bacterial colonization^26^ (mostly ingested). Similarly, acid-reducing drugs reportedly increase bacterial colonization in stomach^28^. This phenomenon correlated with our results that the richness and diversity (Shannon index) of gastric microbiota increased in after-surgery samples. Another potential side effect of vagotomy is cobalamin deficiency, due to decreased gastric intrinsic factor^29^. Consistent with this prediction, our pathway-centric comparison identified decreased abundance of genes for biosynthesis of cobalamin (and other vitamins) after surgery. Therefore, growth of bacteria lacking cobalamin synthesis genes might be the microbial response following vagotomy that contributes to cobalamin deficiency, although it has been proposed that gastrointestinal bacteria in humans are competitors for cobalamin rather than contributors^30^.

In addition to vitamin biosynthesis, other predicted gene functions of differential abundances were analyzed before and after surgery. For example, nitrate and nitrite reductase, phenol degradation gene, and cytochrome were differentially abundant before surgery, all of which are related to bacteria-mediated *N*-nitrosation^22^. It is known that *N*-nitroso compounds are causative factors in carcinogenesis. Therefore, the enrichment of genes functionally associated with *N*-nitrosation before surgery was in agreement with the findings of the cancerous stomach in this study. Since cytochrome commonly presents in respiratory chains and nitrate reductase is used by many Proteobacteria for anaerobic respiration, the differential abundance of these genes was likely a reflection of the high level of Proteobacteria before surgery. As for nitrite reductase, COG1251 (*nirB*) and COG2146 (*nirD*) are functionally associated with nitrate assimilation in various bacteria^31^, suggesting that these two genes are not involved in gastric NO production. To confirm the phenomenon, further experiments are required.

After surgery, the gastric microbiota demonstrated increases in bile salt hydrolase (COG3049 and K01442), NO reductase (COG3256 and COG3901), and N_2_O reductase (COG4263). After Billroth II reconstruction and cholecystectomy (*i.e.*, gallbladder removal), bile salts continuously pass through the stomach (bile reflux) due to anatomical change. This phenomenon speculatively changes the gastric environment in patients after surgery, making the stomach a potential niche for microbes capable of degrading bile salts. This plausibly explains the enrichment of bile salt hydrolase in gastric microbiota after subtotal gastrectomy. Furthermore, NO can be produced enzymatically by activated leukocytes and bacteria^32^ and its bactericidal effect has been suggested to protect the stomach from pathogenic colonization^33^. However, bacterial NO reductase participates in the defense against NO toxicity^34^, implying that the gastric microbiota has a higher capability of NO detoxification after surgery than before surgery. Abundant N_2_O reductase after surgery corresponds to the reported increase in N_2_O concentration after partial gastrectomy^35^, although the microbial effect of N_2_O in stomach is not fully understood.

In conclusion, subtotal gastrectomy alters gastric microbiota in terms of diversity, community composition, and predicted gene functions. These changes in the microbial community of the stomach are closely associated with the altered gastric environment after subtotal gastrectomy.

## Methods

### Study subjects and gastric tissue specimen collection

Gastric tissue specimens were collected from early-stage gastric cancer patients before and after curative subtotal gastrectomy at Taichung Veterans General Hospital. Patients with previous malignancies or who had received chemotherapy, radiation therapy or prior gastric surgery were excluded. In addition, patients who had received proton pump inhibitors, H2 receptor antagonists, antibiotics, or probiotics within 1 month of tissue collection were excluded.

Gastric cancerous tissues and neighboring non-tumor tissues were collected before surgery. All patients underwent subtotal gastrectomy, which included excision of 40–50% stomach, vagotomy, cholecystectomy, and Billroth II reconstruction. Bile flow was through the stomach after surgery due to the method of Billroth II reconstruction. Approximately 2 years after subtotal gastrectomy, gastric tissue specimens were collected from the gastric stump (1 cm away from the anastomosis site) and high body of the lesser curvature of the stomach. Comprehensive oral explanations were given and signed informed consent was obtained from all study subjects. The experiments were carried out in accordance with the protocols approved by the Institute Review Board of Taichung Veterans General Hospital.

### Bacterial genomic DNA extraction

Bacterial genomic DNA was extracted with the Qiagen DNA Mini Kit (Qiagene, MD, USA). Briefly, tissue samples (~20 mg) yielded 15–20 μg genomic DNA for direct use in polymerase chain reaction (PCR) assays and 16S rRNA gene sequencing. Each tissue sample was homogenized by adding lysozyme (100 mg/mL, Sigma-Aldrich, St. Louis, MO, USA) to lysis buffer to promote lysis of Gram-positive bacteria, thereby enhancing total DNA yields. Amount and quality of isolated genomic DNA were determined with NanoDrop ND-1000 (Thermo Scientific, Wilmington, DE, USA). Genomic DNA was stored at –80 °C prior to 16S rRNA sequencing.

### 16S rRNA sequencing and analysis

The hypervariable region V1–V3 of bacterial 16S rRNA genes was amplified by PCR using bar-coded universal primers 27F (F, forward primer; 5′-AGAGTTTGATCMTGGCTCAG-3′) and 534R (R, reverse primer; 5′-GTATTACCGCGGCKGCTG-3′)^36^,^37^. Library construction and sequencing of amplicon DNA samples were conducted with Genomics BioScience (Taipei, Taiwan). A pair-end library (insert size of 490 bp for each sample) was constructed with TruSeq Nano DNA Library Prep kit (Illumina, San Diego, CA, USA) and high-throughput sequencing was performed on an Illumina MiSeq 2000 sequencer with MiSeq Reagent Kit v3 (Illumina).

On a per-sample basis, pair-end reads were merged using USEARCH (v7.0.1090)^38^, with minimum overlap of read pair set at eight base pairs (bp). Merged reads were quality-filtered with Mothur (v1.34.3)^39^ to remove reads shorter than 450 bp or longer than 550 bp, as well as reads with minimum average quality score <27. In addition, reads containing an ambiguous base or homopolymer exceeding 8 bp were excluded. Chimera detection was performed using USEARCH (reference mode and 3% minimum divergence).

Quality-filtered and non-chimeric reads were analyzed (UPARSE^40^ pipeline) to generate OTUs per sample (at 97% identity level). The OTU representative sequences were searched against the Greengenes 13_5 database using USEARCH global alignment to identify the corresponding taxonomy of the best hit. Any OTU without a hit or with only a weak hit, *i.e.* the function “(% sequence identity + % alignment coverage)/2” was <93, was excluded from further analysis. The abundance of each taxon was counted and corrected with PICRUSt^19^, in which the pipeline divided the read count of each taxon by the corresponding 16S rRNA gene copy number. Diversity indices (*e.g.*, Shannon, Simpson, Chao 1, and Good’s coverage) were estimated with Mothur.

### Statistical analysis of bacterial community

All statistical analyses were performed using R software (http://www.r-project.org/), unless otherwise specified. Gene copy number-corrected abundance of genera was total-sum scaled per sample. A pseudocount of 0.0001 was added to the relative abundance (in percentage) before logarithmic transformation^41^. PCA was performed on log-transformed data using the R package ade4^42^ to analyze genera abundance before and after surgery. Between-group inertia percentages were tested (Monte-Carlo test with 10000 permutations) to determine the *P*-values of PCA results. To identify organismal features differentiating communities of stomach bacteria before and after surgery, LEfSe^17^ was applied with α = 0.05 (Kruskal-Wallis and Wilcoxon tests) and effect size threshold of 2 on linear discriminant analysis (LDA) through the web site, http://huttenhower.sph.harvard.edu/galaxy. Community structure similarities within and between groups were assessed using the Bray-Curtis distance^18^ via the R package vegan^43^, based on relative abundance of bacterial genera.

### Prediction and analysis of gene functions of bacterial microbiota

Metabolic profiles of bacterial communities were predicted with PICRUSt, which forecasts abundance of genes of metabolic function based on the 16S copy number-corrected OTU composition. Functional genes were categorized (by PICRUSt) into COGs and KEGG Orthology (KO) gene families. To identify gene functions that differentiated bacterial communities before and after surgery, COG gene abundance was subjected to enrichment analysis of two-group comparison, using the R package ShotgunFunctionalizeR^20^. This analysis normalized gene abundances using a generalized linear model with Poisson canonical logarithmic link function and determined differential significance (*P*-value) via a binomial method with a Benjamini-Hochberg false discovery rate correction to adjust q-values for multiple testing. In addition to gene-centric analysis, ShotgunFunctionalizeR was used to perform pathway-centric analysis. The sets of functionally related COG families were grouped into COG Pathways and COG Categories based on JGI IMG/M^44^. To identify pathways of differential abundance before and after surgery, the two-group comparison of pathways was performed by ShotgunFunctionalizeR using Poisson model to determine differential significance (*P*-value) via a binomial method with a Benjamini-Hochberg false discovery rate correction to adjust q-values for multiple testing.

## Additional Information

**Accession codes:** Illumina pair-end sequencing reads of 24 samples were deposited in the NCBI Sequence Read Archive (accession numberSRP057951).

**How to cite this article**: Tseng, C.-H. *et al*. Gastric microbiota and predicted gene functions are altered after subtotal gastrectomy in patients with gastric cancer. *Sci. Rep.* **6**, 20701; doi: 10.1038/srep20701 (2016).

## Supplementary Material

Supplementary Information

Supplementary Table S2

Supplementary Table S3

## Figures and Tables

**Table 1:** Bacterial community diversity indices based on 16S rRNA gene libraries.

Sample	# OTU^a^*	# SingletonOTU	N^b^	Evenness^c^	Richness^d^*	Shannon	Simpson	Chao 1*	Good’scoverage^e^
T584N	311	158	26306	0.469	35.520	2.694	0.199	766	0.994
T584T	325	158	26306	0.465	35.520	2.692	0.172	837	0.994
T594N	258	158	26306	0.141	35.520	0.785	0.746	829	0.994
T594T	324	210	26306	0.336	47.284	1.944	0.279	888	0.992
T596N	261	158	26306	0.228	35.520	1.269	0.567	849	0.994
T596T	290	184	26306	0.505	41.402	2.863	0.184	939	0.993
T610N	315	184	26306	0.385	41.402	2.212	0.301	824	0.993
T610T	374	210	26306	0.363	47.284	2.152	0.335	949	0.992
T635N	268	184	26306	0.190	41.402	1.063	0.670	849	0.993
T635T	327	210	26306	0.399	47.284	2.312	0.168	970	0.992
T636N	302	184	26306	0.350	41.402	2.001	0.257	1077	0.993
T636T	328	210	26306	0.395	47.284	2.290	0.182	910	0.992
T584S	1580	1105	26306	0.605	249.771	4.453	0.039	6720	0.958
T584B	1517	1105	26306	0.612	249.771	4.481	0.032	7941	0.958
T594S	3219	2815	26306	0.589	636.644	4.754	0.040	36522	0.893
T594B	974	815	26306	0.155	184.161	1.064	0.747	8842	0.969
T596S	1400	1000	26306	0.513	226.015	3.719	0.072	5986	0.962
T596B	1361	947	26306	0.529	214.024	3.817	0.073	5919	0.964
T610S	1199	842	26306	0.549	190.269	3.892	0.072	5631	0.968
T610B	720	447	26306	0.552	100.904	3.635	0.084	2427	0.983
T635S	886	526	26306	0.701	118.777	4.758	0.018	3413	0.980
T635B	911	552	26306	0.655	124.659	4.462	0.034	3251	0.979
T636S	1454	973	26306	0.606	219.907	4.415	0.059	6550	0.963
T636B	925	526	26306	0.608	118.777	4.154	0.077	3086	0.980

^a^OTUs were defined at the 97% sequence identity level, using hypervariable regions V1–V3 of 16S rRNA gene sequences.

^b^The read number was rarefied to the minimum sample size by resampling with 1000 iterations. Data derived from all reads per sample are available (Supplementary Table S1).

^c^Evenness was defined as Shannon/ln(# OTU).

^d^Richness was defined as (# singleton OTU-1)/log_10_N. The maximum value was (N − 1)/log_10_N.

^e^Good’s coverage was defined as 1-(# singleton OTU)/N.

^*^The values of richness indices increased in samples after surgery (*P* < 0.01, one-tailed Student’s *t*-test).
