# Supplementary material for: Gastric microbiota and predicted gene functions are altered after subtotal gastrectomy in patients with gastric cancer
Source: Sci Rep. 2016 Feb 10;6:20701. doi: 10.1038/srep20701 (PMC4748256; doi:10.1038/srep20701)
Supplement: Supplementary Information [file srep20701-s1.pdf]

Supplementary information

Gastric microbiota and predicted gene functions are altered after subtotal gastrectomy in patients with gastric cancer

Ching-Hung Tseng, Jaw-Town Lin, Hsiu J. Ho, Zi-Lun Lai, Chang-Bi Wang, Sen-Lin Tang*, Chun-Ying Wu*

*Corresponding author

Figure

Figure S1 Rarefaction curves of gastric microbiota.

Table

Table S1 Gastric microbiota diversity indices based on 16S rRNA gene libraries.
Table S2 List of COGs enriched in the gastric microbiota before surgery.
Table S3 List of COGs enriched in the gastric microbiota after surgery.
Table S4 Pathways enriched in the gastric microbiota before surgery.
Table S5 Pathways enriched in the gastric microbiota after surgery.

Figure

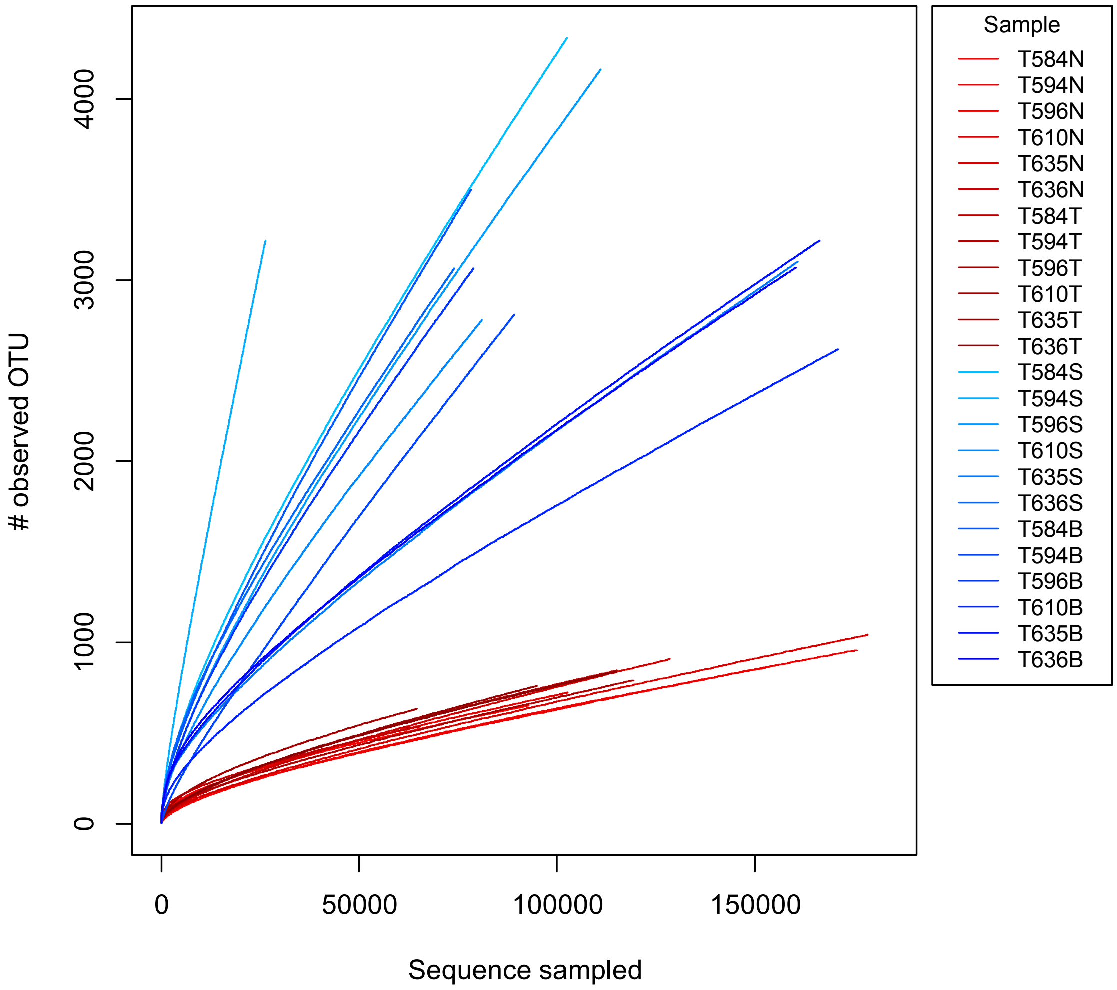

Figure S1 Rarefaction curves of gastric microbiota.

Table

Table S1 Gastric microbiota diversity indices based on 16S rRNA gene libraries.

Sample	# OTU[a]	# Singleton OTU	N	Shannon	Simpson	Chao1	Evenness[b]	Richness[c]	Good's coverage[d]
T584N	651	415	92747	0.417	83.345	2.703	0.199	2441	0.996
T584T	537	318	65454	0.429	65.823	2.699	0.172	1899	0.995
T594N	864	634	153535	0.118	122.055	0.797	0.746	4154	0.996
T594T	845	571	115166	0.290	112.619	1.957	0.278	3015	0.995
T596N	957	725	175779	0.186	138.037	1.279	0.567	5405	0.996
T596T	791	572	119248	0.431	112.480	2.874	0.185	3932	0.995
T610N	723	481	102745	0.338	95.775	2.223	0.301	2943	0.995
T610T	633	393	64558	0.335	81.498	2.160	0.335	2143	0.994
T635N	1064	827	183477	0.154	156.927	1.075	0.670	7274	0.996
T635T	760	526	94884	0.350	105.481	2.324	0.168	3415	0.995
T636N	908	677	128482	0.295	132.320	2.010	0.257	4224	0.995
T636T	838	566	114976	0.342	111.647	2.301	0.182	3298	0.995
T584S	4338	3394	102556	0.539	677.116	4.513	0.039	27370	0.967
T584B	3500	2797	78326	0.555	571.323	4.529	0.032	23656	0.964
T594S	3219	2804	26306	0.589	634.155	4.754	0.040	36522	0.893
T594B	2810	2472	89216	0.139	499.147	1.103	0.747	24625	0.972
T596S	4162	3311	111021	0.453	656.042	3.775	0.072	28409	0.970
T596B	3065	2385	78906	0.480	486.818	3.857	0.073	21646	0.970
T610S	2779	2177	80992	0.496	443.318	3.931	0.073	17962	0.973
T610B	2638	2006	172740	0.465	382.824	3.666	0.084	15956	0.988
T635S	3102	2410	160856	0.597	462.696	4.796	0.018	24928	0.985
T635B	3217	2450	166380	0.557	469.058	4.501	0.034	20659	0.985
T636S	3065	2365	74015	0.555	485.489	4.459	0.059	24735	0.968
T636B	3069	2321	160396	0.522	445.709	4.191	0.077	20782	0.986

[a]OTUs were defined at the 97% sequence identity level using 16S rRNA hypervariable regions V1-V3.
[b]Evenness was defined as Shannon/ln(# OTU).
[c]Richness was defined as (# singleton OTU-1)/$\log_{10}$N. The maximum value is (N-1)/$\log_{10}$N.
[d]Good's coverage was defined as 1-(# singleton OTU)/N.

Table S2 List of COGs enriched in the gastric microbiota before surgery. The results were derived from the enrichment analysis using a gene-centric comparison between predicted gene functional profiles before and after surgery.

Available as separate MS Excel file

Table S3 List of COGs enriched in the gastric microbiota after surgery. The results were derived from the enrichment analysis using a gene-centric comparison between predicted gene functional profiles before and after surgery.

Available as separate MS Excel file

Table S4 Pathways enriched in the gastric microbiota before surgery. The results were derived from the enrichment analysis using a pathway-centric comparison between predicted gene functional profiles before and after surgery.

Pathway category ID	Category size	Gene families found	Coefficient[a]	AIC[b]	Adjusted *P* (BH)	Category
16	17	17	0.026	2245285.676	0	Trypotophan biosynthesis
6	7	7	0.037	3196890.151	0	Pyruvate decraboxylation
17	6	6	0.041	872557.635	0	Valine biosynthesis
49	9	9	0.045	1742493.678	0	Lipid A biosynthesis
70	701	691	0.051	108704817	0	General function prediction only
57	270	270	0.051	50221848.777	0	Amino acid transport and metabolism
67	203	194	0.069	27463190.444	0	Posttranslational modification, protein turnover, chaperones
60	179	179	0.101	27372115.311	0	Coenzyme transport and metabolism
63	230	210	0.112	51839580.910	0	Transcription
7	16	16	0.115	3678208.489	0	TCA cycle
32	16	16	0.140	3912033.215	0	Menaquinone biosynthesis
23	4	4	0.158	585211.766	0	Entner-Doudoroff pathway
14	5	5	0.193	841231.444	0	Proline biosynthesis
73	158	148	0.198	27738034.456	0	Intracellular trafficking, secretion, and vesicular transport
55	258	256	0.206	52309915.139	0	Energy production and conversion
31	14	14	0.209	2593053.542	0	Heme biosynthesis
37	15	15	0.209	3593067.217	0	Ubiquinone biosynthesis
46	69	69	0.234	32688753.934	0	Transcriptional regulators
27	6	6	0.242	703009.960	0	Biotin biosynthesis
72	152	147	0.259	32152847.110	0	Signal transduction mechanisms
30	9	9	0.280	1362369.950	0	FAD biosynthesis
35	7	7	0.299	905013.940	0	Riboflavin biosynthesis
61	94	94	0.461	26072733.063	0	Lipid transport and metabolism
5	15	15	0.518	2230580.452	0	NADH:Ubiquinone oxidreductase subunits
54	19	18	0.520	350586.347	0	Chromatin structure and dynamics
66	96	95	0.563	28142388.630	0	Cell motility
39	13	13	0.601	5870388.272	0	Fatty acid biosyntheis
52	8	8	0.731	624319.101	0	Multisubunit NA+/H+ antiporter
50	33	33	0.762	12203265.587	0	Flagellum structure and biogenesis
69	88	88	0.784	17039862.308	0	Secondary metabolites biosynthesis, transport and catabolism
53	25	22	1.339	643370.497	0	RNA processing and modification
3	2	2	1.432	347301.623	0	Glyoxylate bypass
77	12	9	1.472	282637.313	0	Cytoskeleton
29	9	9	0.025	1326309.589	6.33E-197	Coenzyme A biosynthesis
28	18	18	0.033	2080308.663	9.86E-185	Cobalamin biosynthesis
34	8	8	0.023	1334633.803	8.84E-113	Pyridoxal phosphate biosynthesis
10	6	6	0.020	1154756.679	4.61E-103	Isoleucine biosynthesis
8	11	11	0.014	1973872.842	5.72E-69	Arginine biosynthesis
36	10	10	0.014	2520574.682	3.88E-56	Thiamine biosynthesis

[a]The coefficient is the estimated difference between the two groups (*i.e.*, before and after surgery) using Poisson model. Positive value indicates more abundance before surgery.

[b]The Akaike Information Criterion (AIC) represents a measure of the model fit.

Table S5 Pathways enriched in the gastric microbiota after surgery. The results were derived from the enrichment analysis using a pathway-centric comparison between predicted gene functional profiles before and after surgery.

Pathway category ID	Category size	Gene families found	Coefficient[a]	AIC[b]	Adjusted *P* (BH)	Category
45	15	15	-0.049	2416544.135	0	DNA-dependent RNA polymerase subunits
38	5	5	-0.049	782934.292	0	Deoxyxylulose pathway of terpenoid biosynthesis
68	212	211	-0.057	40662745.992	0	Inorganic ion transport and metabolism
65	188	188	-0.072	44064943.076	0	Cell wall/membrane/envelope biogenesis
13	14	14	-0.077	2064159.447	0	Phenylalanine/tyrosine biosynthesis
2	9	9	-0.077	1276782.166	0	F0F1-type ATP synthase subunits
71	1347	1338	-0.084	126047044.850	0	Function unknown
44	11	11	-0.090	593709.183	0	Basal transription factors
24	14	14	-0.093	2534894.804	0	Gluconeogenesis
40	26	26	-0.098	2953535.462	0	Aminoacyl-tRNA synthetases and alternate systems
15	5	5	-0.121	694831.082	0	Threonine biosynthesis
26	9	9	-0.121	1428890.307	0	Pentose phosphate pathway
22	9	9	-0.129	1376804.125	0	Thymidylate biosynthesis
20	14	14	-0.129	1827467.630	0	Pyrimidine biosynthesis
51	9	9	-0.129	1070802.985	0	Preprotein translocase subunits
56	72	66	-0.139	7219559.122	0	Cell cycle control, cell division, chromosome
64	238	232	-0.187	48914696.065	0	Replication, recombination and repair
41	51	51	-0.189	3824023.524	0	Ribosomal proteins - large subunit
48	8	8	-0.191	1257047.516	0	DNA polymerase III subunits
42	32	32	-0.196	2503329.155	0	Ribosomal proteins - small subunit
43	22	22	-0.198	1251032.039	0	Translation factors and enzymes involved in translation
62	245	242	-0.209	19923262.598	0	Translation, ribosomal structure and biogenesis
59	229	229	-0.214	47873448.357	0	Carbohydrate transport and metabolism
12	10	10	-0.215	1517791.528	0	Methionine biosynthesis
25	14	14	-0.226	3078255.079	0	Glycolysis
74	46	46	-0.233	13431794.355	0	Defense mechanisms
47	26	26	-0.244	2938347.734	0	Basal replication machinery
58	95	95	-0.276	17275668.498	0	Nucleotide transport and metabolism
18	18	18	-0.313	2701319.583	0	Purine biosynthesis
21	10	10	-0.339	2360392.014	0	Pyrimidine salvage
19	5	5	-0.407	1344170.387	0	Purine salvage
1	9	9	-1.321	680246.810	0	Archael/Vacuolar-type H+ ATPase subunits
4	7	7	-2.002	686183.325	0	NA+-transporting NADH:Ubiquinone oxireductase
11	10	10	-0.020	1929078.688	6.85E-135	Leucine biosyntehsis
9	12	12	-0.018	1966818.357	1.11E-85	Histidine biosynthesis
33	7	7	-0.013	911748.5012	6.04E-34	NAD biosynthesis

[a]The coefficient is the estimated difference between the two groups (*i.e.*, before and after surgery) using Poisson model. Negative value indicates more abundance after surgery.

[b]The Akaike Information Criterion (AIC) represents a measure of the model fit.
